# Assessing the Prevalence of HLA-DQ2 and HLA-DQ8 in Lipedema Patients and the Potential Benefits of a Gluten-Free Diet

**DOI:** 10.7759/cureus.41594

**Published:** 2023-07-09

**Authors:** Alexandre C Amato, Lorena L Amato, Daniel Benitti, Juliana L Amato

**Affiliations:** 1 Department of Vascular Surgery, Amato - Instituto de Medicina Avançada, Sao Paulo, BRA; 2 Department of Endocrinology, Amato - Instituto de Medicina Avançada, São Paulo, BRA; 3 Department of Vascular and Endovascular Surgery, Medical Valens Center, São Paulo, BRA; 4 Department of Gynecology, Amato - Instituto de Medicina Avançada, São Paulo, BRA

**Keywords:** hla, hla-dq8, hla-dq2, obesity, lipedema

## Abstract

Objective

The aim of this study is to assess the prevalence of HLA-DQ2 and HLA-DQ8 in women diagnosed with lipedema.

Methods

Leukocyte histocompatibility antigen (HLA) tests of 95 women diagnosed with lipedema were analyzed using non-probabilistic sampling for convenience. The prevalence of HLA-DQ2 and HLA-DQ8 was compared to the general population.

Results

The prevalence of HLA-DQ2+ was 47.4%, that of HLA-DQ8+ was 22.2%, the presence of any celiac disease associated HLA (HLA-DQ2+ or HLA-DQ8+) was 61.1%, both HLA (HLA-DQ2+ and HLA-DQ8+) was 7.4%, and the absence of celiac disease associated HLA was 39%. Compared to the general population, there was a significantly higher prevalence of HLA-DQ2, HLA-DQ8, any HLA, and both HLAs in lipedema patients. The mean weight of patients with HLA-DQ2+ was significantly lower than the overall study population, and their mean BMI significantly differed from the overall mean BMI.

Conclusion

Lipedema patients seeking medical assistance have a higher prevalence of HLA-DQ2 and HLA-DQ8. Considering the role of gluten in inflammation, further research is needed to establish if this association supports the benefit of gluten withdrawal from the diet in managing lipedema symptoms.

## Introduction

Lipedema, primarily characterized by an abnormal accumulation of fat in the legs, often leads to discomfort and a feeling of swelling, particularly when standing [1]. The root cause of lipedema is yet to be clearly understood, but it is known to be linked with inflammation [2,3]. Frequently, lipedema is misdiagnosed as obesity, gynoid lipodystrophy, or lymphedema, leading to its inadvertent oversight in the preliminary stages of medical evaluations. The condition is notably more common in women, and accurate diagnosis can be facilitated using medical imaging techniques such as ultrasound [4], magnetic resonance imaging, and computed tomography. Lipedema presents periodic inflammatory symptoms triggered by various factors, including dietary intake. Interestingly, the inflammation associated with lipedema is not detectable through typical serum markers.

The link between weight gain and the worsening of lipedema symptoms is already well-understood [3]. However, specific foods can aggravate lipedema in ways that extend beyond merely causing weight gain; they can induce heightened inflammation. Consequently, nutrition should play a significant role in lipedema treatment. Past studies suggest a Mediterranean diet approach, which typically recommends a lower carbohydrate intake, around 40% of the total caloric intake, and has proven effective [5]. Additionally, numerous studies have endorsed the benefits of a ketogenic diet [6]. Both these dietary strategies involve reducing or eliminating gluten consumption, and adopting a comprehensive anti-inflammatory approach [7] is also recommended.

Gluten, which comprises around 80% of all proteins found in wheat, plays a significant role in human nutrition [8]. Wheat is a staple in Western diets, commonly consumed as wheat-based or gluten-containing foods. The grain's storage convenience and ability to be easily milled into flour make it a desirable ingredient. It contributes to the texture and flavor appeal of the foods it is included in. Certain studies indicate a potential link between gluten consumption and the surge in non-celiac autoimmune and inflammatory conditions [8]. Ingesting gluten might increase intestinal permeability, facilitating the movement of bacterial lipopolysaccharides and undigested dietary proteins from the gut into the bloodstream through tight junctions. This can trigger an immune and inflammatory response as the body reacts against these substances. Given the concurrent rise in gluten intake and diagnoses of inflammatory conditions such as lipedema, it is crucial to explore more closely the potential association between gluten and lipedema, an inflammatory disorder. Adipose tissue macrophages are shown to play an important role in lipedema, modulating energy metabolism and mitochondrial function [9].

Celiac disease (CD) is an autoimmune disorder directly triggered by gluten consumption [10]. The genes for leukocyte histocompatibility antigens (HLAs) HLA-DQ2 and HLA-DQ8 on chromosome 6p21 are the most significant genetic factors predisposing individuals to CD.

In our clinical experience, we have observed a noticeable improvement in many lipedema patients after they've followed diets that exclude gluten. Various dietary studies [11,12], such as the rare adipose disorder (RAD) diet, which is a modified version of the Mediterranean diet, recommend gluten exclusion despite the lack of a confirmed link with lipedema [13]. Considering the limited research supporting this approach, we decided to examine the frequency of HLA-DQ2 and HLA-DQ8 in lipedema patients without considering inflammatory serum markers.

## Materials and methods

HLA tests of 95 patients were analyzed between August 2022 and May 2023 at Amato Institute's Vascular Surgery Department using non-probabilistic sampling for convenience. We included women diagnosed with lipedema at our institution. The service has a high profile, and patients throughout the region have been referred from primary, secondary, and tertiary care. CD was excluded from all patients in this study by carrying out the following serological tests performed with patients with gluten in their diet: total serum IgA and anti-transglutaminase IgA antibody. Patients younger than 18 years old, as well as those who chose not to participate in the study, were excluded. Obesity alone was not a criterion for exclusion; however, patients who were only obese, without an associated diagnosis of lipedema, were not included in the study.

Diagnosis of lipedema

An expert examiner evaluated the patients in the clinical identification of lipedema. The approach was driven by clinical assessment with diagnostic tests employed in a logical sequence. In our institution, lipedema diagnosis is primarily based on clinical observation (Table 1) and using standardized clinical questions from the QuASiL questionnaire [13]. We use these questions to evaluate the symptoms, comparing their most and least severe instances to determine any improvement in the symptoms. The criteria for classifying a patient as having lipedema include a suggestive clinical history in women after puberty, bilateral and symmetrical accumulation of fat below the hips while sparing the feet (negative Kaposi-Stemmer sign), often non-compressible swelling (negative Godet's sign), tender and sensitive areas to touch, and increased capillary fragility resulting in spontaneous bruising.

**Table 1:** Lipedema clinical characteristics considered in physical examination. The table summarizes the typical features identified during a physical examination and the characteristic signs of lipedema [3].

Characteristics	
Physical examination	Disproportionately increased, loose connective (adipose) tissue on the limbs
	Symmetric tissue
	Palpable tissue nodules beneath the skin
	Tenderness or painful tissue; not always
	Symmetrical limb swelling; pitting or non-pitting
	Hands and feet not affected
	Mobility issues
	Changes in skin texture: thicker or lumpier than normal, similar to an orange peel
Signs	Persistent enlargement of the legs despite elevation
	Easy bruising and/or vascular fragility
	Stemmer-Kaposi sign: inability to pinch and lift the skin on the upper surface of the second toe or finger

We utilized ultrasound criteria to assess and confirm the diagnosis of lipedema [4]. The research employed the Tanita FitScan BC-601 device and dedicated software for bioimpedance analysis.

All patients also had their body mass index (BMI) evaluated. The calculation of limb volume was achieved through the use of bioimpedance. This indirect method estimates volume by evaluating the electrical impedance of different tissues in the body. To make these assessments, it uses varying densities of components such as fat, muscle, bone, and water.

The Tanita scale was used to measure visceral fat. A reading over 13 is considered as having excessive visceral fat.

Statistical analysis

Based on our statistical power analysis, a sample size of 65 patients was calculated as necessary to achieve a 95% confidence interval, considering a 5.5% margin of error for a dichotomous endpoint (HLA-DQ8) and one sample study. However, to further strengthen the study, a total of 95 patients were ultimately included. After manual data consistency verification, descriptive statistical analyses of frequencies were performed using Student's t-test. For the correlations, we assumed a level of statistical significance of 0.05. The software packages used for data analysis were Excel (Microsoft, Redmond, Washington, USA), Wizard 1.9.40 (Evan Miller, Chicago, IL, USA), and MedCalc® Statistical Software version 22.003 (MedCalc Software Ltd, Ostend, Belgium). This study followed the rules of the National Health Council, referring to resolution 196/96 on research involving human beings. It also followed the declaration of Helsinki and was approved by the Institution's Research Ethics Committee (20230105).

## Results

Table 2 summarizes the data for 95 women clinically evaluated in a vascular surgery outpatient clinic and underwent HLA analysis, categorized by the presence of HLA-DQ2, HLA-DQ8, any HLA, no HLA, or both HLAs. The prevalence of HLA-DQ2+, HLA-DQ8+, any HLA (HLA-DQ2+ or HLA-DQ8+), both HLAs (HLA-DQ2+ and HLA-DQ8+), and no HLA was 47.4%, 22.2%, 61.1%, 7.4%, and 39%, respectively. The mean age was 48.85 years (SD±12.41), with no statistically significant difference across the HLA types. We observed differences in weight among the groups. Patients with HLA-DQ2+ had a mean weight of 76.58 kg (SD±11.79, p=0.014), while those with any HLA had a mean weight of 77.69 kg (SD±11.27, p=0.028). Both these groups showed statistically significant differences compared to the overall mean weight of 80.241 kg (SD±12.72). There were also differences in BMI among the groups. Patients with HLA-DQ2+ had a mean BMI of 28.85 kg/m^2^ (SD±4.95, p=0.019) and those with any HLA had a mean BMI of 29.11 kg/m^2^ (SD±4.68, p=0.013), both significantly different from the overall studies subjects mean BMI of 30.27 kg/m^2^ (SD±5.14), while HLA-DQ8 had a mean BMI of 28.98 kg/m^2^. Regarding QuASiL measurements, no statistically significant differences were found across HLA types. Other parameters, including visceral fat and limb volume, showed no significant differences across the groups.

**Table 2:** Comparison of clinical parameters among all patients stratified by HLA-DQ2, HLA-DQ8, any HLA, no HLA, and both HLA statuses. For each category, the number of patients, age, weight, visceral fat, BMI, limbs volume, worst QuASiL, QuASiL variation %, and best QuASiL are provided with corresponding SDs, and p-values were derived from the Student t-test to compare the patients in a specific group against all other patients not included in that particular group. The figures within parentheses represent the percentage of patients or the SD and the p-value for each measurement. BMI, body mass index; SD, standard deviation

	HLA-DQ2+	HLA-DQ8+	Any HLA	No HLA	Both HLAs	All patients
Number of patients	45 (47.4%)	21 (22.2%)	58 (61.1%)	37 (39%)	7 (7.4%)	95
Age (year)	50.12 (SD±13.63, p=0.387)	44.41 (SD±9.23, p=0.1)	48.92 (SD±13.12, p=0.951)	48.75 (SD±11.54)	43.2 (SD±7.62, p=297)	48.85 (SD±12.41)
Weight (kg)	76.58 (SD±11.79, p=0.014)	80.49 (SD±12.24, p=0.69)	77.69 (SD±11.27, p=0.028)	83.77 (SD±13.89)	75,74 (SD±16.88, p=0.418)	80.241 (SD±12.72)
Visceral fat	8.92 (SD±3.39, p=0.272)	8.23 (SD±3.28, p=0.116)	8.88 (SD±3.28, p=0.117)	10 (SD±3.15)	7.4 (SD±12.60, p=0.17)	9.34 (SD±3.26)
BMI (kg/m^2^)	28.85 (SD±4.95, p=0.019)	28.98 (SD±4.83, p= 0.253)	29.11 (SD±4.68, p=0.013)	31.87 (SD±5.37)	27.26 (SD±7.12, p=0.179)	30.27 (SD±5.14)
Limbs volume	26,348 (SD±4,759, p=0.369)	26,754 (SD±4323, p=0.618)	26,671 (SD±4,474, p=0.243)	29,332 (SD±5,670)	25,302 (SD±6,224, p=0.802)	27,776 (SD±5,145)
Worst QuASiL	86.489 (SD±24.67, p=0.722)	95.23 (SD±25.26, p=0.108)	88.94 (SD±25.16, p=0.473)	85.13 (SD±25.20)	98.14 (SD±19.36, p=0.244)	87.46 (SD±25.11)
QuASil variation %	31.87 (SD±21.98, p=0.542)	31.43 (SD±17.01, p=0.661)	32.85 (SD±20.58, p=0.769)	34.27 (SD±23)	22.16 (SD±17.35, p=0.184)	33.41 (SD±21.44)
Best QuASiL	60.84 (SD±25.68, p=0.648)	64.33 (SD±23.78, p=0.368)	59.96 (SD±24.50, p=0.824)	58.66 (SD±28.03)	78.83 (SD±24, p=0.056)	59.45 (SD±5.59)

The prevalence of HLA-DQ2 in our study patients was 47.37% and that of HLA-DQ8 was 22.11% (Table 3). The presence of either HLA-DQ2 or HLA-DQ8 (any HLA) was seen in 61.05% of our study patients. Notably, the prevalence of both HLA-DQ2 and HLA-DQ8 was 7.37%. A chi-squared test indicated that these differences were statistically significant (χ²(3, N = {95}) = 26.128, p < 0.0001). The contingency coefficient was very close to 0, suggesting that the effect size of this association was small.

**Table 3:** Prevalence of HLA-DQ2, HLA-DQ8, any HLA (either HLA-DQ2 or HLA-DQ8), and both HLA (HLA-DQ2 and HLA-DQ8) genotypes in patients with lipedema from our study and in the general population. Percentages indicate the proportion of individuals within each group possessing the specific genotype. (p<0.0001, chi-squared=26.128, degrees of freedom=3, contingency coefficient=0.000337).

	Patients in our study with lipedema	General population
HLA-DQ2	47.37%	41.20%
HLA-DQ8	22.11%	11.30%
Any HLA (HLA-DQ2 or HLA-DQ8)	61.05%	53.70%
Both HLAs (HLA-DQ2 and HLA-DQ8)	7.37%	1.2%

## Discussion

This is the first report of the prevalence of polymorphisms in HLA in a selected cohort of lipedema patients. It is well known that initial lipedema onset can be triggered by hormonal changes such as puberty, pregnancy, and menopause, although the exact cause remains unknown [14]. Additionally, inflammatory symptoms associated with lipedema can be both triggered and alleviated by various factors [2], including dietary approach, where many of them recommend reducing the consumption of carbohydrates, which are rich in gluten. Our study suggests a possible association between the presence of either HLA-DQ2 or HLA-DQ8 - or both - and the inflammation seen in lipedema, especially when combined with gluten intake. However, it is important to clarify that our research does not establish a causative role but merely observes an associative pattern.

CD is an autoimmune disorder characterized by sensitivity to gluten, presenting a wide array of clinical symptoms. A combination of genetic, immunological, and environmental factors causes it. HLA-DQ2 and HLA-DQ8 primarily contribute to its genetic predisposition [15]. Given the disease's strong genetic influence and varying phenotypic expression, a high level of clinical suspicion is often necessary for detection. The disease's pathogenesis involves three key factors: gluten ingestion, changes in intestinal mucosal junctions allowing gliadin to penetrate the barrier and trigger inflammation, and the presence of a specific genetic factor conferred by certain HLAs. In celiac patients with active disease who carry these markers, gluten's interaction with HLA prompts an abnormal immune response in the intestinal mucosa, resulting in tissue damage. However, these alleles are necessary but insufficient for disease development; although the HLA-DQ2 allele is common in the white population, not everyone will develop CD [16]. Individuals who carry the risk alleles have an estimated risk of developing CD ranging from 36% to 53%. This risk is significantly increased before the age of 20 [17].

Among the world's population, the HLA-DQ alleles associated with CD are present in 98.6% of patients with the condition, offering a high negative predictive value. Moreover, in the general population who do not have the diagnosis of CD, HLA-DQ2 and/or HLA-DQ8 is present in approximately 40% of the population, and this percentage increases among patients who are not celiac but have first-degree relatives with CD [15].

While our understanding of the significance of HLA in human diseases has improved significantly, we still need more direct evidence delineating the specific roles of disease-associated HLA molecules and adaptive and innate immunity in developing tissue damage. One study used a mouse model to investigate the connection between HLA positivity and intestinal injury from gluten ingestion. This model simulates the dual overexpression of interleukin-15 (IL-15) in the gut epithelium and the lamina propria, a characteristic feature of active CD. The study demonstrated that CD4+ T cells and HLA-DQ8 are essential for developing villous atrophy, mainly because they are vital in enabling cytotoxic T cells to cause lysis of intestinal epithelial cells. This mouse model, which captures the intricate interaction between gluten, genetic factors, and IL-15-induced tissue inflammation, offers a robust preclinical platform for understanding the cellular mechanisms contributing to intestinal tissue damage in CD [18].

In a cross-sectional study conducted in Brazil, 79.9% of CD patients tested positive for HLA-DQ2, 8% for HLA-DQ8, 10.8% for both HLAs, and 98.6% carried at least one of these HLA types [15]. In contrast, among non-celiac patients without a celiac relative (n=80), 41.2% were positive for HLA-DQ2, 11.3% for HLA-DQ8, 1.2% for both HLAs, and 53.7% carried either HLA [15]. In another study that assessed the prevalence of genetic susceptibility to CD in asymptomatic blood donors in São Paulo, Brazil, 49% of the 404 blood donors tested positive for either HLA-DQ2 or HLA-DQ8, or both [19].

Our study found differences in the prevalence of CD-associated HLA alleles, HLA-DQ2 and HLA-DQ8, in patients with lipedema compared to the general population (Table 3), with a higher prevalence of these HLA alleles in women with lipedema, especially HLA-DQ8. The prevalence of HLA-DQ2 in our study patients was 47.37%, compared to 41.20% in the general population. The prevalence of HLA-DQ8 was also higher in our study group, at 22.11%, compared to 11.3% in the general population. The presence of either HLA-DQ2 or HLA-DQ8 was seen in 61.05% of our study patients versus 53.7% in the general population. Notably, the prevalence of both HLA-DQ2 and HLA-DQ8 was substantially higher in our study group (7.37%) compared to the general population (1.2%). A chi-squared test indicated that these differences were statistically significant (χ²(3, N = {95}) = 26.128, p < 0.0001), suggesting a potential association between these HLA types and lipedema. The contingency coefficient was very close to 0, suggesting that the effect size of this association was small. However, while these findings hint at an association, they do not establish a causal relationship, as per the principles of Mendelian randomization. In the context of human biology, HLA-DR plays a crucial role in the generation and differentiation of adaptive immune responses, and certain HLA class II genes have been linked to an increased susceptibility to various infections and inflammatory diseases. Several studies have also implicated HLA molecules in measures of allergic phenotype and asthma, indicating a potential role in inflammatory responses [20,21]. While a definitive cause-and-effect relationship between HLA-associated inflammation and lipedema has not yet been proven, the results of our study suggest that such a relationship could be plausible.

However, finding a higher prevalence of HLA in our study does not necessarily establish a direct link to lipedema, but it could justify the benefit that these patients have with the removal of gluten from their diet, supporting such a strategy. We observed a significant mean improvement in all patient groups who underwent clinical treatment that included nutritional guidance counseling to remove gluten-rich carbohydrates. This might be due to the fact that the consumption of gluten can increase permeability, potentially leading to an escalation in macrophage activation. Since lipedema has a higher presence of M2 macrophages, this could instigate processes of angiogenesis and fibrosis [9,22,23]. In liposuction, aspiration of lipedemic fat does not address the underlying intolerance, and the surgical procedure itself can stimulate an increase in visceral fat, potentially redirecting the focus of gluten-induced inflammation, further exacerbating brain inflammation. Visceral fat exhibits a stronger correlation with brain atrophy compared to age, BMI, hypertension, and type 2 diabetes mellitus [24]. While this intervention may alleviate limb pain, it does not address the fundamental issue [25-27].

The observed higher prevalence of HLA in lipedema patients underscores the potential benefit of a gluten-free diet in alleviating their symptoms. This particularly applies to patients whose intestinal inflammation could contribute to these symptoms. The results also prompt whether this genetic evaluation might be relevant in other inflammatory diseases, where patients could similarly benefit from eliminating gluten to manage symptoms.

It is important to clarify that our study was not specifically aimed at assessing the improvements resulting from a gluten-free diet in particular patient groups. Those who were already following a gluten-free diet or those who did not adhere to the gluten-free recommendation were not excluded from our research. In our findings, there were no statistically significant differences among these groups. Future studies should investigate whether the improvement of symptoms from a gluten-free diet in lipedema patients is specifically related to individuals who are HLA class gene positive. If further research establishes if this association supports a greater benefit from a gluten-free diet in HLA-positive patients, it could pave the way for more tailored treatment approaches for those with lipedema. Given that patients with more severe, inflamed lipedema often seek medical intervention, and our study exclusively centered around these individuals, we infer that more inflamed lipedema patients seek medical help than those not exhibiting inflammation. Additionally, it is worth noting that asymptomatic lipedema carriers not showing inflammation symptoms may exist, warranting further investigation. Asymptomatic lipedema carriers could present the same HLA proportion as the general population.

Despite its limitations, this study presents an opportunity to delve into the clinical implications of gluten consumption in lipedema patients. Rather than relying on inflammatory lab tests, which can be effectively obscured by lipedema, we chose a different path by focusing on symptomatic, inflamed lipedema. It is important to point out that our study indicates a higher incidence of CD-associated HLA alleles in lipedema patients seeking medical assistance, which corroborates the idea of various inflammatory triggers [2]. Nonetheless, this finding does not establish a causal relationship; CD-associated HLA alleles (HLA-DQ2 and HLA-DQ8) do not appear to be included in the genes necessary for diagnosing lipedema, as 39% of lipedema patients had no such HLA alleles present.

## Conclusions

In the population of lipedema patients seeking medical assistance, there is a significant rise in the prevalence of both HLA-DQ2 and HLA-DQ8. Notably, the increase in the occurrence of HLA-DQ8, and the combined presence of both HLA-DQ8 and HLA-DQ2, is particularly significant. Considering the role of gluten in lipedema patients, further research is needed to establish if this association supports the benefit of gluten withdrawal from the diet in managing lipedema symptoms.

## Appendices

Laboratory Analysis Protocol for Genotyping

DNA for genotyping was extracted from whole venous blood, and an external laboratory conducted the HLA-DQ PCR test to investigate the HLA locus DQ. The DQA1* alleles examined included DQA1*02:EESCV and DQA1*05:EENPV. The DQB1* alleles scrutinized were DQB1*02:EENJU and DQB1*03:EENJV. The test methodology used a medium-resolution PCR-SSOP (polymerase chain reaction with sequence-specific oligonucleotide probe) and a high-resolution NGS (next-generation sequencing).

The HLA-DQ2 heterodimers, which are linked to celiac disease, are as follows: HLA-DQA1*05:01/HLA-DQB1*02:01, HLA-DQA1*05/HLA-DQB1*03:01 in association with HLA-DQA1*02:01/HLA-DQB1*02:02, HLA-DQA1*02:01/HLA-DQB1*02:02 when associated with HLA-DQ8 or particularly applies to dimers. The term "HLA-DQ2 homozygote" refers to HLA-DQ2.5/DQ2.5, HLA-DQ2.5/DQ2.2, or HLA-DQ2.2/DQ2.2. The HLA-DQ8 heterodimer, also associated with celiac disease, is HLA-DQA1*03/HLA-DQB1*03:0.
